# Novel Filaggrin Variants Are Associated with Ichthyosis Vulgaris in Mexicans

**DOI:** 10.3390/genes16040380

**Published:** 2025-03-27

**Authors:** Luz María González-Huerta, Francisco Gabino Zúñiga-Rodríguez, Valeria Isabel Valerio-Gómez, Andrea Aida Velasco-Medina, María del Refugio Rivera-Vega, Edgar Hernández-Zamora, Jaime Toral-López

**Affiliations:** 1 Laboratory of Molecular Biology, Hospital General de México “Eduardo Liceaga” (HGMEL), México City 06720, Mexico; luzma_13_mx@yahoo.com; 2 Department of Medical Genetics, Hospital Regional de Alta Especialidad “Ciudad Salud”, Tapachula 30830, Chiapas, Mexico; tanner_66@hotmail.com; 3 Department of Medical Genetics, Hospital General de México “Eduardo Liceaga” (HGMEL), México City 06720, Mexico; cuqui_rivera@yahoo.com.mx; 4 Department of Immunology and Allergy, Hospital General de México “Eduardo Liceaga” (HGMEL), México City 06720, Mexico; vaiievago12@gmail.com (V.I.V.-G.); a2velascom@gmail.com (A.A.V.-M.); 5 Genomic Medicine, Instituto Nacional de Rehabilitación Luis Guillermo Ibarra Ibarra (INR-LGII), Calzada México-Xochimilco No. 289, Col. Arenal de Guadalupe, Alcaldía Tlalpan, México City 14389, Mexico; 6 Department of Medical Genetics, Centro Médico ISSEMYM Ecatepec, AV del Trabajo S/N, Col. El Carmen, Ecatepec 55000, Estado de México, Mexico

**Keywords:** ichthyosis vulgaris, genodermatosis, filaggrin

## Abstract

Background/Objective: Ichthyosis vulgaris (IV) is a genodermatosis caused by heterozygous, homozygous, or compound heterozygous variants in the filaggrin (*FLG*) gene on chromosome 1q21, which also predispose individuals to atopic dermatitis. Its incidence is 1 in 80–250 children. The phenotypic characteristics include palmar hyperlinearity, keratosis pilaris, and a fine scale that is most prominent over the lower abdomen, arms, and legs. Our objective was to study the genetic variants in the *FLG* gene and their associations in patients with ichthyosis vulgaris. Material and methods: Here, we studied eighteen Mexican sporadic cases and four family members with IV. Steroid sulfatase (STS) enzymatic activity, polymerase chain reaction (PCR), and direct sequencing on the *FLG* gene were conducted. Results: We found the recurrent heterozygous variant R501* in fifteen sporadic cases, while the other three sporadic cases showed four novel (p.Q2123R, p.H2118R, p.D2120E, p.S3970L) variants and one reported (p.Y2119H) variant; members of family 1 and 2 presented novel homozygous and heterozygous (p.S1482Y, p.P2144S) variants. Conclusions: This study added to the novel pathogenic variants in patients with IV and showed that the stop mutations (p.R501*) in the Mexican population are the most prevalent.

## 1. Introduction

Ichthyoses is a heterogeneous group of disorders associated with alterations in the keratinization of skin, which are characterized by hyperkeratosis and/or scaling. In 2009, a classification was described based mainly on clinical characteristics, but considering the pathophysiological and molecular aspects. This classification identified 36 types of ichthyoses, which were divided into subgroups according to presence or absence of extracutaneous involvement, the frequency of the disease, and the inheritance pattern. Extracutaneous manifestations allow for the identification of two large groups of ichthyoses. Non-syndromic ichthyosis is when genetic defects manifest exclusively in the skin, while syndromic ichthyosis is defined by the presence of alterations in other organs or systems besides the skin. These alterations can be inherited or acquired [1]. The most common type of ichthyosis is ichthyosis vulgaris, which accounts for more than 95% of ichthyosis cases. It presents an autosomal dominant inheritance pattern and incomplete penetrance. It generally begins in childhood, but is not present at birth, in contrast to congenital ichthyosis, and continues to affect the individual throughout life. Ichthyosis vulgaris (IV; OMIM: # 146700) is the most common type of hereditary ichthyosis related to the filaggrin gene (*FLG*; OMIM: # 135940), followed by the X-linked recessive ichthyosis (XLI; OMIM: 308100) which is related to the steroid sulfatase gene (STS; OMIM: # 300747) [2,3].

IV is characterized by hyperkeratosis and/or desquamation. It is one of the most frequent single gene disorders in humans with an incidence of 1 in 80–250 children [4,5,6]. Among the phenotypic characteristics are palmar hyperlinearity, keratosis pilaris, and a fine scale that is most prominent over the lower abdomen, arms, and legs. The histology is characterized by mild ortho-hyperkeratosis and a reduced or absent stratum granulosum [7].

**Figure 1:**
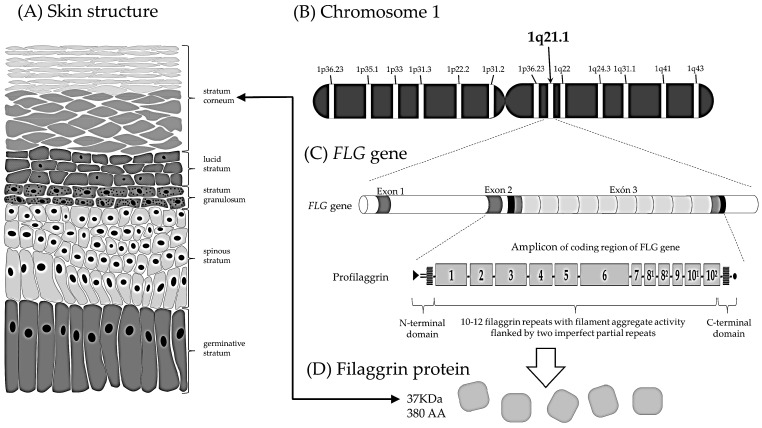
*FLG* expression in skin. (**A**) The structure of the different layers of skin, with FLG being particularly expressed in the stratum corneum. (**B**) The *FLG* gene is located on chromosome 1, at locus 1q21. (**C**) Profilaggrin is encoded by the *FLG* gene and consists of three exons. (**D**) Filaggrin is a protein found in the skin that is essential for the formation of the skin barrier.

IV can be caused by heterozygous, homozygous, or compound heterozygous variants in the gene encoding the filaggrin (*FLG*) (NCBI/Uniprot: P20930) protein, located on chromosome 1q21 (Figure 1) [2,8,9,10]. Prevalent *FLG* gene mutations are R501*, 2282del4, R2447*, and S3247*; these may be associated with atopic dermatitis in 37–50% of cases [11,12,13]. There are various investigations of variants, mutations, and polymorphisms of associated patients with IV in Asians, Europeans, and North Americans, among others [14,15,16,17,18,19].

FLG is a structural protein which is produced in keratinocytes. It plays an essential role in the organization of keratin filaments and in the development of the stratum corneum of the skin. FLG has 4061 amino acids (aa) [18] and is initially synthesized as a large, insoluble, highly phosphorylated precursor called profilaggrin containing many tandem repeated copies of 324 aa, which are separated by small linker coding sequence, and is the main component of the keratohyalin granules of the granular layer of the epidermis. FLG corresponds to approximately 6% of the protein content of the epidermis (Figure 1) [20,21].

The *FLG* gene has three exons: exon 1 consists only of 15 bp (5′ UTR region); exon 2 (159 bp) contains the initiation codon; and exon 3 is very large (12,753 bp) and encodes ten filaggrin repeats, two partial or imperfect repeats, and the N-terminal domain (Figure 1). Loss-of-function mutations in *FLG* are the cause of IV and are major genetic predisposing factors for atopic dermatitis (AD). Several null mutations in the *FLG* gene that lead to a decrease or absence of filaggrin in skin and predispose individuals to these conditions have been described [19].

X-linked recessive ichthyosis (XLI) is a hereditary skin disease with a prevalence of 1/6000 to 1/2000 in males and without any ethnic or geographical differences. The causative gene for XLI is the steroid sulfatase gene (*STS*), located on Xp22.3. Further understanding the role of the *STS* gene pathogenic variants in XLI may contribute to a more accurate and efficient clinical diagnosis of XLI and provide novel strategies for its treatment and prenatal diagnosis [22].

Based on this background, our objective was to study genetic variants in the *FLG* gene and their associations in patients with a clinical diagnosis of ichthyosis vulgaris.

## 2. Material and Methods

### 2.1. Participants

This investigation began with approximately 2100 Mexican patients diagnosed with nonspecific ichthyosis. As a result of a detailed clinical diagnosis, 200 patients were identified with a clinical diagnosis of IV. A total of 22 cases from 200 with ichthyosis vulgaris were included in this report; four family cases (in two families) and eight non-family cases (NF). A clinical history was taken, which included family history, current condition, complete physical examination, and the patients were diagnosed using the clinical criteria for IV. One hundred healthy individuals (controls) of both sexes were also included (data no show). All participants were of legal age. A blood sample was taken from each participant and collected in a tube with EDTA K2. All hemolyzed or lipemic samples were discarded.

### 2.2. STS Activity

STS activity was determined in leukocytes as follows: 10 mL of blood was obtained with a heparinized syringe. The leukocyte pellet was obtained through centrifugation and washed three times with 0.9% NaCl. STS assay was performed in the leukocyte pellet, which was homogenized in chilled 0.014 M Tris (hydroxymethyl-aminomethane buffer) with a polytron in two cycles which lasted 20 s and 10 s, respectively. 7-[^3^H]-dehydroepiandrosterone sulfate (16.3 Ci per mmol, NEN, Boston, MA, USA) was used as the enzyme substrate. The assay conditions were pH 7.0 at 37 °C for 1 h in a final volume of 250 mL of 0.014 M Tris buffer. The product of hydrolysis was recovered with benzene (analytical grade, Merck, Darmstadt, Germany) and read in a scintillation spectrometer. Each essay was performed twice with similar results and a normal control was always included [23].

### 2.3. Automated Sequencing

Genome DNA was extracted from the leukocytes in peripheral blood using a commercial kit Puregene (Qiagen, Hilden, Germany), as per the manufacturer’s protocol. After genome DNA extraction, the *FLG* gene analysis was carried out by polymerase chain reaction (PCR) and DNA direct sequencing. The PCR amplicon of exon 3 of the *FLG* gene was subjected to a sequencing reaction using 2 mL of amplicon annealed with 2 mL of big dye v3.1, under the following conditions: 96 °C for 10 s, 55 °C for 15 s, and 68 °C for 2 min, for 30 cycles in a 2720 thermal cycler (Thermo Fisher Scientific, Waltham, MA, USA). The PCR sequence product was purified using Centrisep columns with Sephadex G-50, molecular biology grade, and was analyzed by capillary electrophoresis using the ABI3500 sequencer (Thermo Fisher Scientific, Waltham, MA, USA). The conditions and primers to amplify an amplicon of the coding region of the *FLG* gene were previously described (Figure 1). PCR products obtained from the cases and controls were sequenced on an ABI 3500 Automated Sequencer (PE Biosystems, Foster City, CA, USA) [24].

### 2.4. Ethical Aspects

All participants were selected under the guidelines of the Norma Oficial Mexicana NOM-253-SSA1-2012 for blood banks [25]. All participants were of legal age, received oral and written information about the study, and signed a letter giving consent in writing to participate in this study.

This study was conducted according to the Declaration of Helsinki principles, and was approved by the Research, Biosafety and Ethics Committees of Hospital General de México “Eduardo Liceaga” (approval code: DI/23/501/04/32; approval date: 21 August 2023).

## 3. Results

In the clinical diagnosis of IV, the physical examination showed that all patients presented typical erythematous scabby lesions, appearing as bright, intense red erythema, with parchment-like, pinkish skin, and on their skin surface they presented abundant, large, grayish scales that detached easily. In addition, STS activity was determined. All patients with IV had normal STS activity levels (0.11–0.92 pmol/mg protein/h), which completely ruled out a diagnosis of XLI.

Directed sequencing analysis revealed the variants in 22 cases with IV. Four of them were family cases (two families). Family 1 comprised a father (IVF1f) and daughter (IVF1d), Family 2 a mother (IVF2m) and daughter (IVF2d), and the remaining 18 corresponded to non-family cases (NF) (Table 1).

**Figure 2:**
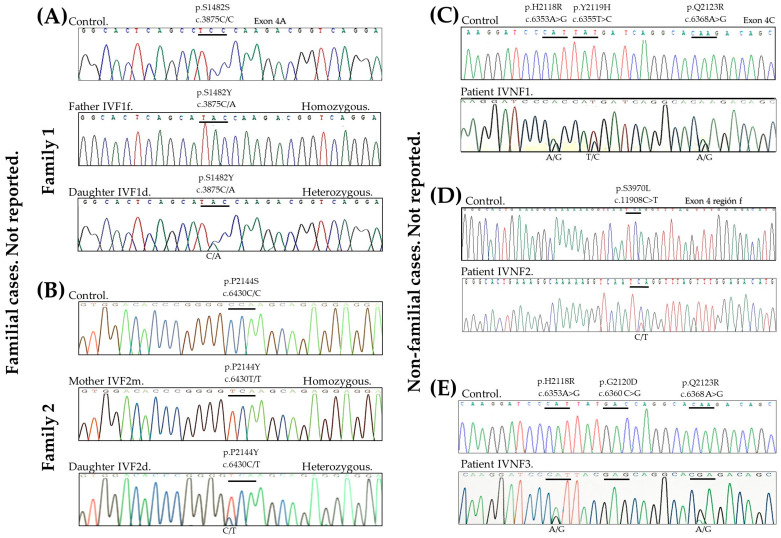
Electropherograms of familial cases and controls: (**A**) Family 1 and (**B**) Family 2. Electropherograms of non-familial cases and controls: (**C**) Patient IVNF1. (**D**) Patient IVNF2, and (**E**) Patient IVNF3.

Figure 2 shows the electropherograms of the sequences of the patients that presented novel mutations compared with their respective controls. The novel mutations found are described in Table 1. The 15 NF (IVNF4 to IVNF18) cases presented the mutation R501*. This is a recurrent mutation that has already been described by Smith et al. in 2006 [5].

## 4. Discussion

A descriptive, prospective study was carried out in twenty-two patients with a clinical diagnosis of IV; there were four family cases and eighteen non-family cases.

In the family cases, we found that two members of Family 1 presented a variant of novel single nucleotide (SNV) in a homozygous or heterozygous state (p.S1482Y). Family 2, similarly, also presented novel SNV in a homozygous or heterozygous state (p.P2144S). These SNVs have the molecular consequence of missense, which results in a nucleotide change, resulting in the replacement of an amino acid. However, until now, according to the record described in the publicly accessible database on genetic changes and their relationship to human health, ClinVar (CV), no association with any pathology has been reported (CV. Record last updated 13 January 2025). Therefore, this is the first time that these new SNVs have been linked to Mexican patients with IV.

Regarding the non-family cases, in three of them, undescribed variants were found in the filaggrin gene in the homozygous or heterozygous allelic state (p.Q2123R, p.H2118R, p.S3970L, and p.D2120E).

In one of three non-family cases (participant record IVNF1), we found the variant p.Y2119H. When searching for this variant, we found that a similar variant, p.Y2119* described by Margolis et al. in African American patients with atopic dermatitis (AD), was an FLG loss-of-function (LoF) variant that was not previously identified [26]. However, p.Y2119H (CV ID 1221916) has been described as missense.

Similarly, in cases with participant records IVNF1 and IVNF3, the novel variant p.Q2123R was located at the same amino acid site as a previously reported variant p.Q2123* in Indian patients with AD, which is also related to FLG LoF [26]. However, the p.Q2123R variant in particular has also not been reported. It is important to remember that FLG LoF variants are associated with IV and the major genetic risk factors for developing AD [16].

Also, in patient IVNF1, the H2118R variant was found, which is new, since it has not yet been described in any other study. Similarly, there were two other new mutations, D2120E (also present in patient IVNF3) and S3970L which were present in patient IVNF2 (CV ID 1231239). Also, this has been described as missense, but both variants have also not been reported in another study.

Finely, the remaining 15 non-family cases (IVNF4 to IVNF18) presented the heterozygous variant R501*, already reported and associated with IV previously by Smith et al. [5]. In this population of Mexicans with IV, it was the most prevalent mutation.

Our patients did not show evidence of AD. In The Human Gene Mutation Database (HGMD), about 80 variants in the *FLG* gene have been reported, 71 being nonsenses and 9 being missenses. In general, nonsense variation causes a protein to stop or terminate translation earlier than expected, resulting in a shorter protein, which sometimes results in a change or loss of function. In contrast, a missense variation results in an amino acid change in the resulting protein, which may or may not alter its function. Filaggrin deficiency disrupts corneocyte differentiation and the formation of natural moisturizing factor, leading to hyperkeratosis due to water retention and increased transepidermal water loss. In our patients, one nonsense and seven missense and variants were observed, all related to IV. The R501* variant was in the first repeat region; p.S1482Y was in the fourth repeat region; p.H2118R, p.Y2119H, p.G2120D, p.Q2123R, and p.P2144Y were in the sixth repeat region; and p.S3970L was in the distal partial.

## 5. Conclusions

We found the recurrent change R501* in fifteen sporadic patients, while the other six novel variants were detected in three sporadic cases and in the four members of the two families. This study shows that the stop mutation (p.R501X) is the most prevalent in the Mexican population with IV. In addition, these results added novel variants to the diversity of mutations found on the *FLG* gene.

## Figures and Tables

**Table 1:** Filaggrin variant’s location in FLG associated with ichthyosis vulgaris and the characteristics of each of the mutations found in the *FLG* gene (1q21.3) in Mexican patients.

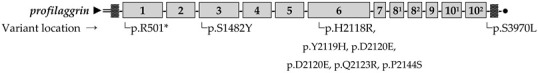							
Family Cases	Participant Record	Protein	Nucleotide	Control	Change Allele Found in Patient	CV Variation ID	References
Family 1	IVF1f father	p.S1482Y	c.4445C>A	TCC	T**A**C, T**A**C	1277512	NR
	IVF1d daughter			TCC	TCC, T**A**C		
Family 2	IVF2m mother	p.P2144S	c.6430C>T	CCA	**T**CA, **T**CA	1206192	NR
	IVF2d daughter			CCA	CCA, **T**CA		
No	IVNF1	p.H2118R p.Y2119H p.Q2123R	c.6353A>G c.6355T>C c.6368A>G	CAT TAT CAA	CAT, C**GC** TAT, **C**AT CAA, C**G**A	NR 1221916 NR	NR Margolis, 2019 [26] NR
No	IVNF2	p.S3970L	c.11908C>T	TCA	TCA, T**T**A	1231239	NR
No	IVNF3	p.H2118R p.D2120E p.Q2123R	c.6353A>G c.6360C>G c.6368A>G	CAT GAC CAA	CAT, C**G**T GAC, GA**G** CAA, C**G**A	NR NR NR	NR NR NR
None	IVNF4 to IVNF18	p.R501*	c.1501C>T	CGA	**T**GA, **T**GA	16319	Smith FJ, 2006 [5]

IV: ichthyosis vulgaris. F: family cases. f: father. d: daughter. m: mother. NF: non-family cases. All variants found in the ClinVar page are as follows: effect: missense and clinical significance: not reported. CV: ClinVar. ID: identificator. NR: not reported. ► S100 calcium binding domain. ═ B-domain. 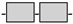 *FLG* repeats. ▓ Imperfect *FLG* repeats. ● C-terminal domain.

## Data Availability

The original contributions presented in the study are included in the article, further inquiries can be directed to the corresponding author.
